# Northern Ohio Dental Association

**Published:** 1869-06

**Authors:** Henri L. Ambler

**Affiliations:** Youngstown


### Proceedings of Societies.

### NORTHERN OHIO DENTAL ASSOCIATION.

YOUNGSTOWN, May 4th, 1869, 11 A. M.

The Association met according to the requirements of the Constitution.

President Dr. W. P. Horton in the Chair.

Minutes of the last annual meeting read and approved.

Names of members present: B. Strickland, C. R Butler, F. S. Slosson, B. T. Spelman, Corydon Palmer, A. E. Lyman, W. P. Horton, J F. Siddell, L. Buffett, C. C. Carroll, F. S. Whitslar, C. Buffett, J. G. Templeton, H. L. Ambler, D. R. Jennings, E. A. Way.

The Executive Committee reported the following order of exercises for this session:

Reading the minutes of the last meeting.

Report of Officers.

Report of Committees.

Election of Officers.

Election of members.

Election of delegates to the American Dental Association.

Essays and Discussions.

C. BUFFETT,<br>F. S. WHITSLAR, } *Committee.*

The report of the Treasurer was read by the Chair, and referred to Dr. Siddell, as auditor.

The Committee on Incorporation reported that there was no law in this State whereby such an association as this could be incorporated.

F. S. SLOSSON,<br>W. P. HORTON,<br>C. R. BUTLER, } *Committee.*

The election of officers for the ensuing year resulted in choice of the following:

*President*—F. S. Whitslar.

*Vice-President*—L. Buffett.

*Recording Secretary*—H. L. Ambler.

*Corresponding Secretary*—C. Buffett.

*Treasurer*—C. R. Butler.

*Board of Examiners*—L. Buffett, C. R. Butler, Corydon Palmer.

On motion, the association adjourned until 2 P. M.

### FIRST DAY—AFTERNOON SESSION.

The association met according to adjournment. President, Dr. Horton, in the chair.

Minutes of the last meeting read and approved.

Dr. Whitslar, the incoming President, was then conducted to the chair by Drs. Spelman and Buffett.

The President then remarked that he was under obligations to the association for this new honor which they had seen fit to bestow upon him, although he was fearful he did not merit it; he should at all times try and do his duty, and judging by the goodly number present, it is deemed a good sign that the Dental fraternity are alive and glad to learn all that may be new and can be imparted by their brother members. Once more I thank the association for this mark of their approbation, and in accepting the duties of the office hope I may be able to conduct our meetings with impartiality and justice to all.

Dr. Horton, the retiring President, upon retiring remarked: A bright day seems to be coming for the Dental profession in Ohio, as the law regulating the Practice of Dentistry has been passed, thus shutting out quacks from the full liberty which they before enjoyed; also, many other great improvements have been made in our specialty. We still hope that this association will work harmoniously, as they have done heretofore.

Dr. Horton offered the following resolution:

*Resolved*, That the Dentist from Kent is a proper person for active membership in this association, provided he has been in practice in this State for the last year, and provided that he pass the necessary examination.

Dr. Horton further remarked: Dental Education is a special education, and has for its object the elevation of two classes, the Dental operator and the generality of mankind. There is connected with it, something more than a mere mechanical pursuit. Both the hand and the head must be educated, as is well known by all scientific men. There are also many sciences to be studied in connection with Dentistry, such as Anatomy, Physiology, Pathology, etc. These underlie the profession of which we are the students. In our Dental colleges, preparations are complete for teaching all these branches, and one who desires to enter the profession, can do so now, at the point where years ago it would have taken very much more time to attain the same proficiency. Now we have a systematized course of study, where we can get both the theory and the practice; these advantages have been by untiring zeal arrived at. Our journals are multiplying, and these are a great source of information, containing many articles connected with our profession that are very instructive, and have cost the writers many hard hours of labor. Many physicians have treated patients systemically, when they should have been treated locally; all, because they had not been schooled in our specialty. Have noticed that in the Mississippi Valley there is a deficiency of lime substance in the tooth structure, and especially is this so in children. Both the parent and the child must be educated how to care for their teeth, then the time will soon come when people will have better teeth; for the Dentist and physician will have a better knowledge of each others specialties.

Dr. Butler then read an Essay on Contour Fillings, which was very instructive, and gave many new ideas worthy of imitation, as they were good and useful.

On motion, the association adjourned until 7½ P. M.

FIRST DAY—EVENING SESSION.

The association met according to adjournment.

President, Dr. Whitslar, in the chair.

Reading of minutes postponed.

The subject of Contour Fillings was then discussed by Drs. Strickland, Buffett, Palmer and others. A voluntary essay, subject, "The Dentist," was read by Dr. Siddell. It was somewhat novel, striking home at many points. The subject was discussed by Drs. Spelman, Slosson, Palmer, and others.

An essay was then read by Dr. Corydon Palmer, subject, Extraction of the Teeth, in connection with their method of preservation. The teeth should seldom be extracted, only when nothing can be done to preserve them. Those who make artificial dentures their specialty, sacrifice thousands of teeth which could be preserved; thus depriving their patients of that which they can never restore, for the purpose of enriching themselves; striving to make their patients believe they are giving them better teeth than those nature provided. But the people are not so much to blame for this general destruction, it is upon the Dentist that the reflection is cast; for with the daily evidence they have before them, they must know that the teeth can be restored in size and form, being rendered beautiful and useful. In these days, there can be no excuse for Dentists not qualifying themselves, so as to be able to preserve these natural organs. Let all try and see how many perfect gold fillings they can make, restoring the size and form of the tooth, and rendering them useful, instead of tearing them from their sockets to make room for artificial dentures. The Dentist must instruct the people in the care of their teeth. and by this means help bring about a change for the better. One and all, begin with new courage, and strive to do all the good we can by saving these important organs.

The essay was received and placed on file. Remarks on the essay were made by Drs. Butler, Slosson, and others.

On motion, the association adjourned until 8½ A. M.

SECOND DAY--MORNING SESSION.

The association met according to adjournment.

President, Dr. Whitslar, in the chair.

Minutes of last meeting read and approved.

Dr. STRICKLAND offered the following amendment to the Constitution:

*Resolved*, That it shall be the duty of the Secretary to strike out from the list of active members, the name of any person who has failed to participate in the meetings of the association and pay his dues to the same for two successive years, and the person so expelled shall not be restored again to membership, except by a vote of two-thirds of all the members present at any regular meeting.

An Essay on Dental Hygiene was read by H. L. Ambler.

On motion, it was received and placed on file.

Remarks were then made on the subject by Drs. Butler, Buffett, and Palmer.

Drs. Butler, Templeton, Ambler, Palmer and Whitslar, were appointed delegates to the American Dental Association.

Dr. Carroll read an Essay on Alveolar Abscess. It was discussed by Drs. Spelman, Ambler and others.

On motion, the association adjourned until 1½ P. M.

SECOND DAY—AFTERNOON SESSION.

Meeting called to order by the President.

On motion, Drs. Templeton, Jennings and Ambler, the Committee on Dental Ethics was continued for one year.

The chair appointed as Executive Committee for the ensuing year, Drs. Buffett, Horton and Spelman.

Dr. Way exhibited cases of rubber work made after Dr. Stucks' Patent, involving the use of metallic dies for vulcanizing upon.

The Committee on Dental Ethics reported as follows: That S. B. Burnham be cited before this Committee to answer specific charges for unprofessional conduct. That C. B. Knowlton be cited before this Committee to answer these charges,—1st. Advertising to perform work at extremely low prices, in order to injure competitors. 2d. For inserting teeth in the mouth on plates which he admits are poisonous. That A. E. Lyman, in the opinion of your Committee, is unable to refute the charges brought against him of unprofessional conduct; and he is hereby expelled from the Association.

Report accepted and adopted.

On motion, H. L. Ambler was appointed a delegate at large, to European Societies.

On motion, the Association adjourned to meet at Cleveland, in one year.

HENRI L. AMBLER,
*Recording Secretary.*

